# Associations of patient knowledge with drug-modifiable cardiovascular risk factor control in coronary artery disease patients with and without diabetes mellitus: results from the cross-sectional KNOW-ABC study

**DOI:** 10.1186/s12872-025-04599-7

**Published:** 2025-03-05

**Authors:** Maximilian Brockmeyer, Michaela Fell, Claudio Parco, Alexander Hoss, Kris G. Vargas, Emilia Wies, Yingfeng Lin, Yvonne Heinen, Nadja Chernyak, Andrea Icks, Christian Jung, Malte Kelm, Georg Wolff

**Affiliations:** 1 Department of Internal Medicine, Division of Cardiology, Pulmonology and Vascular Medicine, Medical Faculty and University Hospital Düsseldorf, Heinrich Heine University Düsseldorf, Düsseldorf, Germany; 2 Nuffield Department of Population Health, University of Oxford, Oxford, UK; 3 Institute for Health Services Research and Health Economics, Centre for Health and Society, Medical Faculty and University Hospital Düsseldorf, Heinrich Heine University Düsseldorf, Düsseldorf, Germany; 4 Institute for Health Services Research and Health Economics, German Diabetes Center, Leibniz Center for Diabetes Research at the Heinrich Heine University Düsseldorf, Düsseldorf, Germany; 5 CARID – Cardiovascular Research Institute Düsseldorf, Düsseldorf, Germany; 6 Clinic for Cardiology, Klinikum Ibbenbüren, Ibbenbüren, Germany; 7 Department of Internal Medicine, Division of Cardiology, Pulmonology and Vascular Medicine, Medical Faculty and University Hospital Düsseldorf,, Heinrich Heine University Düsseldorf, Moorenstr. 5, 40225 Düsseldorf, Germany

**Keywords:** Patient knowledge, Patient information, Treatment goals, Low-density lipoprotein cholesterol, Blood pressure, Glycated hemoglobin, Coronary artery disease

## Abstract

**Background:**

Control of major drug-modifiable risk factors for glycated hemoglobin (HbA1c), blood pressure (BP), and low-density lipoprotein cholesterol (LDL-C) remains unsatisfactory in the secondary prevention of coronary artery disease (CAD). We aimed to analyze patient knowledge and attainment of LDL-C, BP, and HbA1c treatment goals and associated factors in German CAD patients with and without diabetes mellitus (DM).

**Methods/Results:**

A total of 204 CAD patients (68 ± 8 years; 75.0% male; 84 with DM (41.2%)) completed a questionnaire assessing their knowledge of LDL-C (< 55 mg/dL), BP (age-adapted), and HbA1c (< 7.0%) treatment goals and levels of information on predefined CAD topics as well as associated factors, including CAD duration, adherence to pharmacotherapy, and physician monitoring of secondary prevention. LDL-C, BP, and HbA1c were measured. The mean duration of CAD was 9.8 ± 8 years. A total of 98.5% reported good adherence to pharmacotherapy. Measurements of LDL-C (81.4%) and HbA1c (71.4%) were predominantly performed by general practitioners. LDL-C goals were attained significantly better in patients with DM (39.3% with vs. 16.7% without DM, *p* < 0.01). The attainment of BP goals did not differ between patients with and without DM (71.4% vs. 72.5%, *p* = 0.87). HbA1c goals were attained by 48.8% of DM patients. LDL-C goals were known by 6.0% of patients with vs. 9.2% without DM (*p* = 0.44), and BP goals were known by 36.9% with vs. 30.0% without DM (*p* = 0.36). Knowledge of HbA1c goals was prevalent in 53.6% of DM patients. Subjective levels of information on CAD topics did not differ between patients with and without DM. Logistic regression revealed that DM (odds ratio (OR) 3.73, 95% confidence interval (CI) 1.82–7.63) and knowledge of treatment goals were associated with LDL-C goal attainment (OR 3.84, CI 1.19–12.41); no such associations were identified for BP or HbA1c.

**Conclusions:**

In German CAD patients with and without DM, a remarkable lack of knowledge and attainment of LDL-C treatment goals exists compared with BP and HbA1c. DM and knowledge of treatment goals were significantly associated with LDL-C treatment goal attainment. General practitioners rather than cardiologists or other specialties currently manage risk factor control.

**Trial registration:**

German Clinical Trials Register studyID DRKS00030703.

**Supplementary Information:**

The online version contains supplementary material available at 10.1186/s12872-025-04599-7.

## Introduction

Atherosclerotic cardiovascular disease (ASCVD), especially coronary artery disease (CAD), is the leading cause of death worldwide and ranks first in health expenditures in developed countries [1, 2]. Hence, cardiovascular risk modification is of paramount medical, social, and economic importance in secondary CAD prevention. Low-density lipoprotein cholesterol (LDL-C), blood pressure (BP), and glycated hemoglobin (HbA1c) are the most important pharmacologically modifiable risk factors. In large cardiovascular outcome trials, pharmacological interventions to reduce LDL-C, BP, and HbA1c have improved the outcomes of patients with cardiovascular disease [3–5]. Subsequently, international cardiovascular guidelines promote precise treatment goal strategies for each risk factor to ensure adequate control [6–8]. However, treatment goal attainment remains poor in real-world settings, with considerable differences between risk factors [9, 10]. 

The successful implementation of guideline-recommended secondary preventive pharmacotherapy to attain treatment goals is challenging: There are several multilayered, interacting factors on the physician and patient sides, as well as factors related to physician‒patient interactions and external regulations to consider [11]. Different physician specialties manage risk factor control in patients with CAD: general practitioners and cardiologists are primarily involved; other specialties, e.g., diabetologists in patients with diabetes mellitus (DM), also contribute.

Current guidelines give the highest class of recommendation to an informed discussion about cardiovascular risk and treatment benefits with the patient, which necessitates an assessment of the patient’s disease-related knowledge and respective information needs [12]. A previous work involving a cohort of patients with ASCVD and DM [13] reported a remarkable deficit in knowledge of LDL-C treatment goals and subjective levels of disease-related information on ASCVD and LDL-C goal attainment, especially compared with knowledge and attainment of HbA1c goals. Additionally, patients felt better informed about topics related to DM than ASCVD. Owing to methodological limitations, the study was unable to detect associations of patient knowledge with the attainment of treatment goals.

In the present work, we thus aimed to extend our investigations to a general population of persons with CAD to analyze attainment and patient knowledge of LDL-C and BP treatment goals, with a focus on differences between patients with and without DM. We explored subjective levels of information on the topics of CAD as well as possibly associated factors of treatment goal attainment, including disease duration, adherence to pharmacotherapy and the specialties of physicians managing risk factor control.

## Methods

### Study design, screening and patient selection

Between July and December 2022, we conducted a cross-sectional study (German Clinical Trials Register study-ID: DRKS00030703) in patients hospitalized in a general ward at Düsseldorf Heart Center in Germany. Patients ≥ 18 years of age were eligible after providing written informed consent if they were previously diagnosed with CAD. The exclusion criteria were suspected or diagnosed cognitive impairment, a language barrier and ongoing intravenous antihypertensive treatment. The study was positively evaluated by the ethics committee of the Medical Faculty of Heinrich Heine University Düsseldorf (Study No. 2022–1907) and conducted in accordance with the ethical standards of the Declaration of Helsinki.

### Data assessment and treatment goal definitions

Patient characteristics, including comorbidities, history of cardiovascular events, and ongoing cardiovascular and glucose-lowering pharmacotherapy, were obtained from medical records. Peripheral venous blood was collected to assess LDL-C and HbA1c serum levels. BP was measured at rest right after study inclusion via an automated clinical digital sphygmomanometer.

The LDL-C goal of < 55 mg/dL was defined for all patients as recommended by the 2019 ESC guidelines [6]. BP goals were applied according to the 2018 ESC guidelines [7], with goals for systolic BP < 130 mmHg in patients < 65 years of age and < 140 mmHg for elderly patients ≥ 65 years. For all patients, the goal for diastolic BP was < 80 mmHg. The HbA1c treatment goal was defined according to 2019 ESC guidelines: [8] the primary goal of HbA1c was < 7.0%; a secondary goal of HbA1c < 8.0% was evaluated separately for elderly patients ≥ 65 years of age.

### Questionnaire

A questionnaire (in German language) designed by a multidisciplinary team of researchers, positively evaluated by external specialists in diabetology and lipidology, and used in previous work served as the basis for the questionnaire of the present study [13]. 

For assessment of objective knowledge of treatment goals, we asked the participants to name their assumed treatment goals for LDL (mg/dL), BP (mmHg), and HbA1c (%). The participants could state the value or answer “*I don’t know”*.

To assess subjective levels of disease-related information and information needs on topics of CAD, we utilized an adapted version of the Information Needs in Diabetes Questionnaire that was previously extended to ASCVD [13, 14]. Predefined disease-related topics of interest were *cause of the disease*, *course of the disease*, *long-term complications*, *treatment/therapy*, *lifestyle adjustment*,* health promotion and information sources (lifestyle adjustment*,* etc.)*, and *support*, *helpline and information sources*. Subjective levels of information were measured on a 4-point Likert scale (*very well*, *well*,* not well*, and *not informed at all*). Additionally, patients were asked to state the need for additional information on every topic (*yes* or *no*).

The medical specialty of physicians managing risk factor control was investigated. We asked patients which physician specialty primarily performed laboratory analyses of LDL-C and HbA1c (possible answers: no analyses performed, general practitioner, cardiologist, other specialty, or unknown). In a second step, patients were asked to attribute responsibility for risk factor control of LDL-C and HbA1c to a physician specialty (possible answers: general practitioner, cardiologist, other specialty, patient, or unknown).

Moreover, patients were asked to report the time since diagnosis of CAD (years) or could answer *“I don’t know*”; likewise, patients with DM were asked about the time since the diagnosis of DM.

Self-reported participation preferences in medical decision-making were assessed via the Control Preference Scale and coded by *passive role*, *collaborative role* and *active role* [15]. In addition, the highest educational degree reported by patients was recorded. Patient-reported general adherence to pharmacotherapy was measured by the Rief Adherence Index (RAI) [16]. Good adherence to pharmacotherapy in general was defined as a score of ≤ 8 according to the RAI [16]. 

The questionnaire was distributed to participants during their hospital stay and was collected the same day. The questionnaire translated into English is available in the Supplementary.

### Statistics

Given the lack of evidence of patient knowledge on LDL-C, BP, and HbA1c treatment goals and the lack of disease-related information in the general CAD population, we conducted an explorative, hypothesis-generating study. We set a sample size of approximately *n* = 200 to be sufficient. Continuous data are presented as the means ± standard deviations, and ordinal/categorical data are presented as counts and percentages of the total. In the case of missing data, this is indicated accordingly, and the number of patients included in the specific analysis is evident. Contingency analyses of dichotomous outcomes of knowledge and respective attainment of treatment goals were performed via chi-square tests and Fisher’s exact tests. Dichotomous outcomes of paired data were compared via McNemar’s test. The results of all six individual 4-point Likert items were summed for each participant to compare overall subjective levels of disease-related information between patients with and without DM via a two-sided unpaired t test. Data analysis was performed via SPSS 23.0 (IBM) and GraphPad Prism 7.0.

The possible factors associated with the attainment of LDL-C, BP, and HbA1c treatment goals were analyzed via binary logistic regression. Variable selection included sociodemographic and clinical factors (age, sex, highest level of education, DM, and disease duration) and was based on previous studies reporting associations with the attainment of treatment goals [13, 17–19]. Knowledge of treatment goals, summed subjective levels of information, and participation preferences were additionally included because of the assumption of an association with increased patient awareness of secondary preventive treatment. Additionally, the variable “physician specialty primarily responsible for risk factor control” was selected to explore the impact of regulatory healthcare factors.

Statistically significant differences in any test result were assumed at a two-sided *p* < 0.05.

## Results

### Sample characteristics

The sociodemographic and clinical characteristics of the study population are displayed in Table 1. Among the 204 CAD patients who were included (mean age 68 ± 8 years, 71.4% male), 84 (41.2%) had previously been diagnosed with DM, 73 (35.8%) had a history of myocardial infarction, 176 (86.3%) had a percutaneous coronary intervention, and 45 (23.5%) had previously undergone coronary bypass surgery. The mean time since the diagnosis of CAD was 9.8 ± 8 years. The mean duration of DM was 15.1 ± 10 years since diagnosis (data from six patients were missing). Most patients had a previous diagnosis of arterial hypertension (91.7%). Compared with patients without DM, those with DM were more likely to have chronic kidney disease (estimated glomerular filtration rate ≤ 60 ml/min; 48.8% vs. 31.7%, *p* = 0.01), a history of stroke (11.9% vs. 3.3%, *p* = 0.02), and peripheral artery disease (39.3% vs. 20.0%, *p* < 0.01). The majority of patients reported a lower secondary education degree (69.1%; International Standard Classification of Education level 2); fewer patients with DM reported having a university degree (International Standard Classification of Education level ≥ 6; 11.9% vs. 23.3%, *p* = 0.04). With respect to participation preference in medical decision-making, a *passive role* was most commonly preferred (40.0% without DM vs. 46.4% with DM, *p* = 0.36).

**Table 1:** Patient characteristics

Baseline characteristics	Total (*n* = 204)	With DM (*n* = 84)	Without DM (*n* = 120)	*p*-value
Age (years)	68 ± 8	66.6 ± 8	68.2 ± 7	0.16
BMI (kg/m²)	28.0 ± 5	28.7 ± 5	27.6 ± 6	0.13
Male	153 (75.0%)	68 (81.0%)	85 (70.8%)	0.11
Active smoker	48 (23.5%)	21 (25.0%)	27 (22.5%)	0.68
CAD	204 (100%)			
Duration (years)	9.8 ± 8	9.6 ± 8	9.74 ± 8	0.92
Myocardial infarction	73 (35.8%)	33 (39.3%)	40 (33.3%)	0.38
Percutaneous coronary intervention	176 (86.3%)	73 (86.9%)	103 (85.8%)	0.83
Coronary bypass surgery	45 (23.5%)	24 (28.6%)	24 (20.0%)	0.16
Heart failure with reduced ejection fraction	35 (17.2%)	18 (21.4%)	17 (14.2%)	0.18
Arterial hypertension	187 (91.7%)	78 (92.9%)	109 (90.8%)	0.61
DM	84 (41.2%)	84 (100%)	-	
Duration (years)*	15.1 ± 8	15.1 ± 8	-	
Type 1	6 (2.9%)	6 (7.1%)	-	
Type 2	78 (38.2%)	78 (92.9%)	-	
Cerebral artery disease	9 (4.4%)	5 (6.0%)	4 (3.3%)	0.49
Stroke	14 (6.9%)	10 (11.9%)	4 (3.3%)	**0.02**
Peripheral artery disease	57 (27.9%)	33 (39.3%)	24 (20.0%)	**< 0.01**
Chronic kidney disease (eGFR ≤ 60 ml/min)	79 (38.7%)	41 (48.8%)	38 (31.7%)	**0.01**
Highest level of education				
University degree	38 (18.6%)	10 (11.9%)	28 (23.3%)	**0.04**
Higher secondary degree	19 (9.3%)	7 (8.3%)	12 (10.0%)	0.68
Lower secondary degree	141 (69.1%)	64 (76.2%)	77 (64.2%)	0.07
No degree	6 (2.9%)	3 (3.6%)	3 (2.5%)	0.58
Patient participation preference				
*Active role*	57 (27.9%)	26 (31.0%)	31 (25.8%)	0.41
*Collaborative role*	60 (29.4%)	19 (22.6%)	41 (34.2%)	0.75
*Passive role*	87 (42.6%)	39 (46.4%)	48 (40.0%)	0.36

Patient characteristics of all included patients (*n* = 204). Additionally, the characteristics of patients with (*n* = 84) and without diabetes mellitus (*n* = 120) are reported separately. Data are presented as n (%) or as the mean ± standard deviation; * data from 6 patients were missing. BMI = body mass index; CAD = coronary artery disease; DM = diabetes mellitus; eGFR = estimated glomerular filtration rate

### Characteristics of secondary preventive pharmacotherapy

In terms of lipid-lowering therapy, no significant differences were found between patients with and without DM (Table 2): 91.2% were on prescriptions of any statin (95.3% patients with DM vs. 88.3% without DM, *p* = 0.09), 58.3% were on high-intensity statin therapy (atorvastatin ≥ 40 mg/day or rosuvastatin ≥ 20 mg/day; 61.9% patients with DM vs. 55.8% without DM, *p* = 0.39), and 24.5% were on a combination of any statin and ezetimibe (29.8% with DM vs. 20.8% without DM, *p* = 0.15). The prescription of novel lipid-lowering agents such as bempedoic acid, proprotein convertase subtilisin/kexin type 9 (PCSK9) inhibitors or inclisiran has rarely been reported (2.5% of all patients).

**Table 2:** Treatment characteristics

Treatment	Total (*n* = 204)	With DM (*n* = 84)	Without DM (*n* = 120)	*p*-value
Lipid-lowering therapy				
Any statin	186 (91.2%)	80 (95.2%)	106 (88.3%)	0.09
High-intensity statin	119 (58.3%)	52 (61.9%)	67 (55.8%)	0.39
Ezetimibe	53 (26.0%)	25 (29.8%)	28 (23.3%)	0.30
Statin + ezetimibe	50 (24.5%)	25 (29.8%)	25 (20.8%)	0.15
Bempedoic acid	2 (1.0%)	-	2 (1.7%)	-
PCSK9-inhibitor	2 (1.0%)	-	2 (1.7%)	-
Inclisiran	1 (0.5%)	1 (1.2%)	-	-
Antihypertensive therapy				
ACE-inhibitor / angiotensin-receptor blocker	168 (82.4%)	65 (77.4%)	103 (85.6%)	0.12
Betablocker	179 (87.7%)	73 (86.4%)	106 (88.3%)	0.76
Dihydropyridine calcium channel blocker	59 (28.9%)	25 (29.8%)	34 (28.3%)	0.83
Diuretic	122 (59.8%)	62 (73.8%)	60 (50.0%)	**< 0.01**
Mineralcorticoid receptor antagonist	46 (22.5%)	24 (28.6%)	22 (18.3%)	0.09
Diabetes mellitus therapy				
Metformin	42 (20.6%)	42 (50.0%)	-	-
GLP-1 receptor agonist	5 (2.5%)	5 (6.0%)	-	-
SGLT2 inhibitor	65 (31.9%)	44 (52.4%)	21 (25.0%)	**< 0.01**
DPP-4 inhibitor	19 (9.3%)	19 (22.6%)	-	
Sulfonylurea	2 (1%)	2 (1%)	-	-
Insulin	40 (19.6%)	40 (19.6%)	-	-
Antiplatelet/anticoagulant therapy	202 (99%)	82 (97.6%)	120 (100%)	0.09
Good adherence (patient-reported; Rief adherence index ≤ 8)	201 (98.5%)	94 (100%)	117 (97.5%)	0.14

Characteristics of cardiovascular and diabetes mellitus pharmacotherapy in all included patients (*n* = 204). Additionally, the characteristics of patients with (*n* = 84) and without diabetes mellitus (*n* = 120) are reported separately. The data are presented as n (%). GLP-1 = glucagon-like peptide-1; SGLT2 = sodium/glucose cotransporter 2; DPP-4 inhibitor = dipeptidyl peptidase-4 inhibitor; ACE = angiotensin converting enzyme; PCSK9 = proprotein convertase subtilisin/kexin type 9

A total of 82.4% of all patients received an angiotensin converting enzyme (ACE)-inhibitor or angiotensin-receptor blocker (77.4% of patients with DM vs. 85.6% without DM, *p* = 0.12), 87.7% received a beta blocker (86.4% of patients with DM vs. 88.3% without DM, *p* = 0.76), and 28.9% received a dihydropyridine calcium channel blocker (29.8% of patients with DM vs. 28.3% without DM, *p* = 0.83). Patients with DM received diuretics more frequently than those without DM did (73.8% vs. 50.0% with DM, *p* < 0.01; Table 2).

In patients with DM, 50.0% were on the prescription of metformin, 6.0% were on a glucagon-like peptide-1 (GLP-1) receptor agonist, 52.4% were on a sodium‒glucose cotransporter 2 (SGLT2) inhibitor, and 22.6% were on a dipeptidyl peptidase-4 (DPP-4) inhibitor. Insulin therapy was carried out in 19.6% of patients with DM (Table 2).

Good adherence to pharmacotherapy according to the RAI was reported by 98.7% (100% with DM vs. 97.5% without DM, *p* = 0.14).

### Treatment goal attainment

The mean LDL-C serum level was significantly lower in patients with DM than in those without DM (69.5 ± 29.7 mg/dL vs. 81.4 ± 36.1 mg/dL, *p* = 0.01; 76.5 ± 34.0 mg/dL in all patients). The mean BP was 125/71 ± 21/11 mmHg in all patients, without significant differences between patients with and without DM (125/71 ± 20/10 mmHg vs. 124/71 ± 24/12 mmHg, *p* = 0.66). In patients with DM, the mean HbA1c was 7.1 ± 1.3% (5.6 ± 0.5% in patients without DM).

**Fig. 1:**
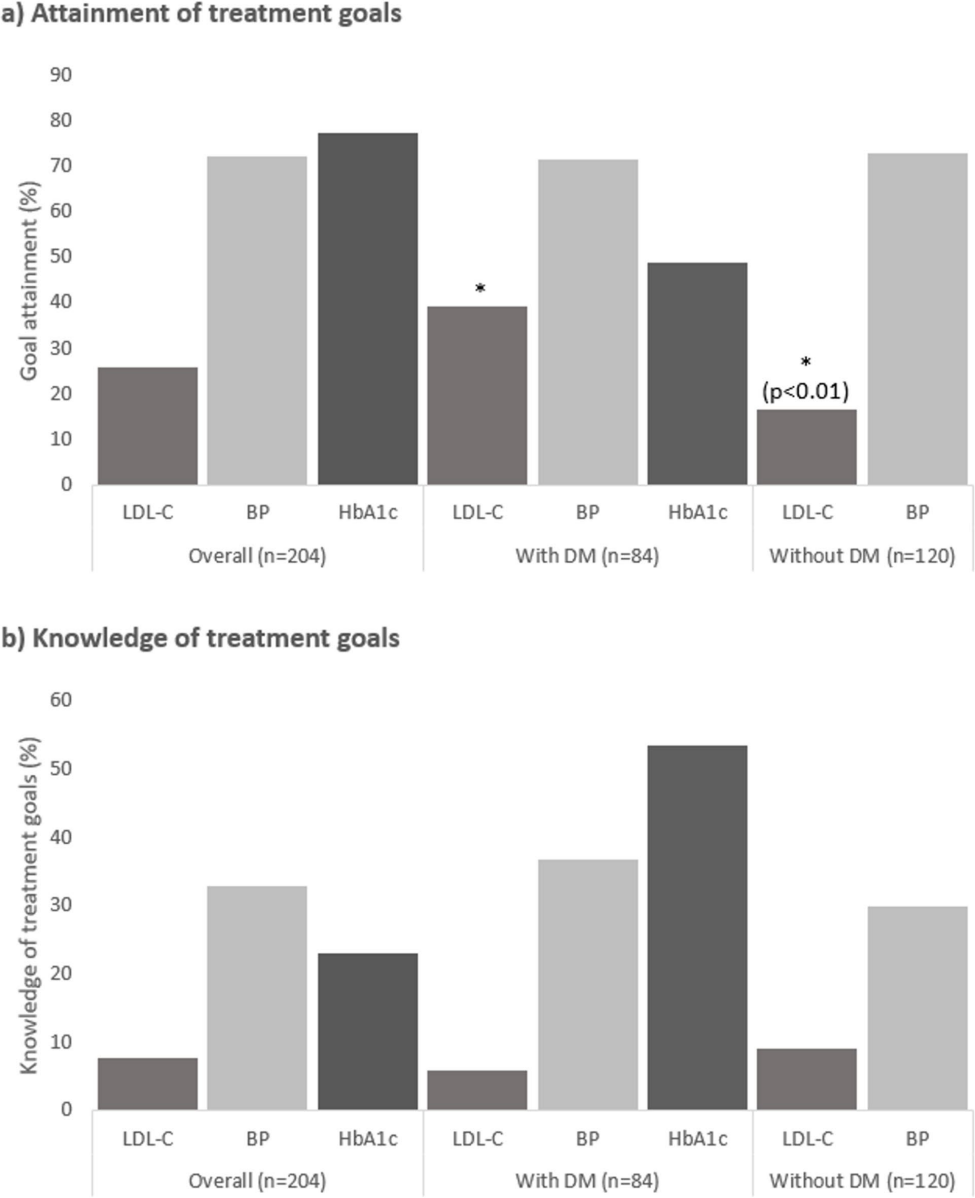
Attainment and knowledge of treatment goals.**(a)** Attainment of treatment goals (in %) in all patients (*n* = 204) and in patients with (*n* = 84) and without diabetes mellitus (DM, *n* = 120) in terms of low-density lipoprotein cholesterol (LDL-C), blood pressure (BP), and glycated hemoglobin A1c (HbA1c, patients with DM only). **(b)** Objective knowledge of treatment goals (in %) in all patients (*n* = 204) and in patients with (*n* = 84) and without DM (*n* = 120) of LDL-C, BP, and HbA1c (patients with DM only)

Figure 1a displays attainment of LDL-C, BP, and HbA1c treatment goals: LDL-C treatment goal of < 55 mg/dL was attained inadequately overall (26.0%), however, more frequently by patients with DM than those without (39.3% vs. 16.7%, *p* < 0.01). Age-adapted treatment goals for BP were attained by 72.1% of all patients; no differences between patients with and without DM were observed (71.4% vs. 72.5%, *p* = 0.87). A total of 48.8% of patients with DM achieved the HbA1c goal. The HbA1c level was < 7.0% in 97.5% of patients without known DM. Consequently, at least three patients with undiagnosed DM were identified. Additional analyses for systolic and diastolic goals, as well as treatment goals of HbA1c < 8.0% for elderly patients ≥ 65 years, are listed in Supplementary Table 1.

Comparing goal attainment between risk factors revealed that BP goals were attained more frequently than LDL-C goals (72.1% vs. 26.0% for all patients, 71.4% vs. 39.3% for patients with DM, and 72.5% vs. 16.7% for patients without DM; p for all comparisons < 0.01). In patients with DM, BP goals were attained more frequently than HbA1c goals (71.4% vs. 48.8%, *p* < 0.01), without differences in HbA1c vs. LDL-C goals (48.8% vs. 16.7%, *p* = 0.23). When additional treatment goals of HbA1c < 8.0% were applied for elderly patients ≥ 65 years, the HbA1c goal was attained in significantly more patients than the LDL-C goal (69.0% vs. 39.3%, *p* < 0.01; Supplementary Table 1).

### Knowledge of treatment goals and subjective levels of disease-related information

The questionnaire was completed by all 204 patients. Knowledge of LDL-C goals was found in 7.8% of all patients (6.0% with DM vs. 9.2% without DM, *p* = 0.44), knowledge of BP goals was found in 32.8% of all patients (36.9% with DM vs. 30.0% without DM, *p* = 0.36), and knowledge of HbA1c goals was found in 53.6% of patients with DM (1.7% in patients without DM; Fig. 1b).

Accordingly, among patients with DM, the proportion of patients with knowledge of HbA1c goals was significantly greater than those with BP (*p* = 0.02) and LDL-C goals (*p* < 0.01; Fig. 1b). In contrast, among those without DM, significantly more patients could name the correct BP goal than the LDL-C goal (*p* < 0.01; Fig. 1b).

**Fig. 2:**
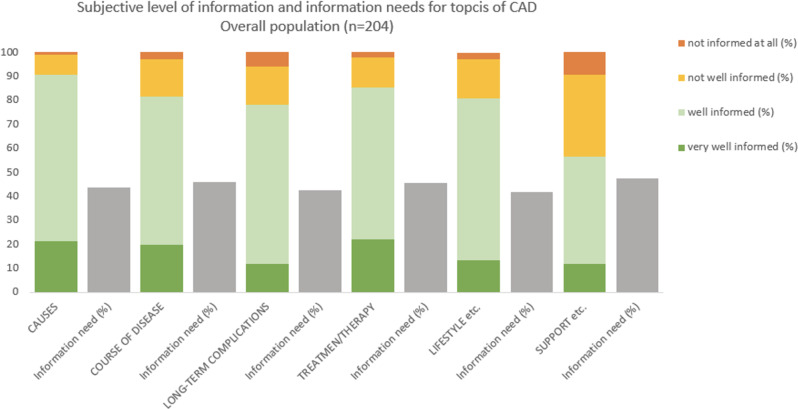
Subjective level of disease-related information and information needs. Graphical display of the subjective level of disease-related information and the subjective need for more disease-related information on topics related to coronary artery disease (CAD) in the overall population (*n* = 204). The subjective level of information was measured on a 4-point Likert scale (*very well*,* well*,* not well*,* and not informed at all*). Additionally, patients were asked to state the need for additional information on every topic (*yes* or *no*)

The subjective levels of information and information needs of the overall population on the topics of CAD are displayed in Fig. 2. The highest levels of information were found for the topic of *cause of the disease*, without differences for patients with and without DM (91.6% vs. 90.0% *very well* or *well informed*, *p* = 0.27). The lowest (although still relatively high) levels of information were found for *support*, *helpline*,* and information sources* (51.2% *very well* or *well informed* with DM vs. 60.0% without DM, *p* = 0.42). An overall comparison of summed subjective levels of information on CAD topics revealed no differences between patients with and without DM (mean summed score of answers to all topics on a 4-point Likert scale 17.3 with DM vs. 17.6 without DM, *p* = 0.44; Supplementary Table 1).

Among all patients, information needs were highest for the topic of *support*, *helpline and information sources* (47.5%), without significant differences between patients with and without DM: 44.0% with DM compared with 50.0% of patients without DM wished to receive more information on this topic (*p* = 0.40; Fig. 2 and Supplementary Fig. 1). The lowest need for information was reported for *lifestyle adjustment* (41.7% overall; 39.3% with DM vs. 43.3% without DM, *p* = 0.56; Fig. 2 and Supplementary Fig. 1). For all other topics assessed by the questionnaire (*cause of the disease*,* course of the disease*, *long-term complications*, *treatment/therapy*, and *support*, *helplines*,* and information sources*), information needs did not significantly differ between patients with and without DM (p for all > 0.1; Supplementary Fig. 1).

### Management of risk factor control: medical specialties

Patients reported that risk factor control (i.e., laboratory measurements of LDL-C and HbA1c and respective therapy adjustments) was predominantly performed by their general practitioner (LDL-C: 85.1% general practitioner; 11.2% cardiologist, 3.6% other specialties; HbA1c: 76.9% general practitioner, 8.6% cardiologist, 14.2% other specialties; Supplementary Table 2).

With respect to HbA1c, more patients without DM reported that no measurements of HbA1c were performed prior to study inclusion than DM patients did (95.2% with DM vs. 30.8% without DM, *p* < 0.01; Supplementary Table 2).

In addition, patients most frequently attributed responsibility for risk factor control to their general practitioner (LDL-C: 75.0% general practitioner; 16.7% cardiologist, 3.4% other specialty, 2.9% patient, 2.0% unknown; HbA1c: 73.5% general practitioner, 5.9% cardiologist, 9.8% other specialty, 1.5% patient, 9.3% unknown; Supplementary Table 3).

### Associated factors of treatment goal attainment

Multivariate logistic regression identified factors associated with LDL-C goal attainment: coexisting DM (odds ratio (OR) 3.73, 95% confidence interval (CI) 1.82–7.63; *p* < 0.01), a lower level of subjective disease-related information for CAD (OR 1.18, CI 1.04–1.35; *p* = 0.01), and knowledge of LDL-C treatment goals (OR 3.84, CI 1.19–12.41; *p* = 0.02; Table 3). Age, sex, duration of CAD, highest level of education, participation preferences, and specialty of physician performing LDL-C and HbA1c control were not associated with attainment treatment goals (Table 3).

**Table 3:** Factors associated with the attainment of HbA1c, BP, and LDL-C treatment goals

Attainment of LDL-C treatment goals^a^			
Variable	Odds ratio	95% confidence interval	*p*-value
Age (years)	1.01	0.97–1.05	0.74
Sex (male/female)	0.70	0.30–1.65	0.41
Duration of CAD (years)	0.98	0.94–1.03	0.42
DM	3.73	1.82–7.63	**< 0.01**
Highest level of education (no > lower secondary > higher secondar > university degree)	1.18	0.87–1.59	0.29
Summed subjective level of information on topics of CAD (1–24 points)	0.85	0.74–0.96	**0.01**
Knowledge of LDL-C treatment goal	3.84	1.19–12.41	**0.02**
Participation preferences (active > collaborative > passive role)	0.87	0.57–1.32	0.52
Measurement of LDL-C: General practitioner	0.94	0.39–2.28	0.89
**Attainment of BP treatment goals** ^**b**^			
**Variable**	**Odds ratio**	**95% confidence interval**	**p-value**
Age (years)	1.02	0.98–1.06	0.36
Sex (male/female)	1.15	0.55–2.44	0.71
Duration of CAD (years)	1.02	0.98–1.07	0.29
DM	1.07	0.56–2.04	0.84
Highest level of education (no > lower secondary > higher secondar > university degree)	1.20	0.90–1.60	0.21
Summed subjective level of information on topics of CAD (1–24 points)	1.05	0.95–1.18	0.35
Participation preferences (active > collaborative > passive role)	0.93	0.64–1.36	0.71
Knowledge of BP treatment goal	1.15	0.58–2.28	0.68
**Attainment of HbA1c treatment goals (patients with DM)** ^**c**^			
**Variable**	**Odds ratio**	**95% confidence interval**	**p-value**
Age (years)	0.99	0.93–1.05	0.72
Sex (male/female)	2.73	0.77–9.69	0.12
Duration of CAD (years)	0.94	0.87–1.01	0.71
Duration of DM (years)	0.97	0.93–1.03	0.06
Highest level of education (no > lower secondary > higher secondary > university degree)	0.75	0.46–1.23	0.25
Summed subjective level of information on topics of CAD (1–24 points)	1.00	0.83–1.20	0.96
Knowledge of HbA1c treatment goal	0.76	0.25–2.31	0.62
Participation preferences (active > collaborative > passive role)	0.81	0.44–1.51	0.51
Measurement of HbA1c: General practitioner	1.71	0.48–6.12	0.41

Factors associated with attainment of low-density lipoprotein cholesterol (LDL-C), blood pressure (BP), and glycated hemoglobin A1c (HbA1c) treatment goals according to multivariate logistic regression (*n* = 210). For patients with diabetes mellitus (DM) (*n* = 78), six of the 84 patients with DM were excluded because of missing data on the duration of DM. CAD = coronary artery disease; ^a^Hosmer-Lemeshow for goodness of fit of the model *X*^*2*^ *= 3.68*,* df =* 8, p *=* 0.89; ^b^Hosmer-Lemeshow for goodness of fit of the model *X*^*2*^ *= 11.59*,* df =* 8, p *=* 0.17; ^c^Hosmer-Lemeshow for goodness of fit of the model *X*^*2*^ *= 18.34*,* df =* 8, p *=* 0.19

The same analysis did not identify factors associated with BP or HbA1c treatment goal attainment. For analysis of factors associated with HbA1c goal attainment in patients with DM, six of 84 patients with DM were excluded because data on the duration of DM were missing (Table 3).

## Discussion

In this cross-sectional study, we investigated risk factor knowledge with drug-modifiable risk factor control in CAD patients with and without DM. The main findings are as follows: (1) knowledge of HbA1c and BP treatment goals was found more frequently than knowledge of LDL-C treatment goals, in conjunction with goal attainment; (2) knowledge of LDL-C goals was associated with LDL-C goal attainment, which was not found for HbA1c and BP; (3) patients with DM attained LDL-C treatment goals more frequently than patients without DM; and (4) general practitioners rather than cardiologists or other specialties predominantly perform risk factor management of LDL-C and HbA1c.

Risk factor control by optimal medical therapy in patients with ASCVD and CAD is of paramount importance for improving patient outcomes, especially since the benefits of percutaneous coronary interventions in chronic coronary syndrome patients have been questioned by the results of RCTs in recent years [20, 21]. However, the results of the present study and data from international large-scale registries show poor rates of attaining secondary preventive treatment goals, with considerable differences between risk factors. Whereas BP goals were attained by approximately 70% of CAD patients, HbA1c goal attainment was found in 45–60% of CAD patients [9, 22, 23]. LDL-C goal attainment of approximately 20% in the present study and in general CAD populations certainly indicates a unsatisfactory state of control of a risk factor [24], which is acknowledged as causal for the pathogenesis and progression of CAD [25]. Given that several drug classes, such as high-intensity statins [26], ezetimibe [27], bempedoic acid [28], and PCSK9 inhibitors [29, 30], improve patient outcomes and are capable of lowering LDL-C to target patients, health services research on LDL-C risk factor management is urgently needed to find measures to ensure that patients actually receive and adhere to guideline-recommended lipid-lowering therapy.

Risk factor management entails a complex process that involves patient‒physician interactions and factors that affect patients and physicians individually [11, 23]. With respect to patients, we identified an alarming deficit in knowledge of LDL-C goals (< 8%, Fig. 1b) compared with HbA1c and BP goals in CAD patients with and without DM, which is similar to the results of a previous study in a cohort of DM patients with ASCVD [13]. Similarly, we again found a remarkable discrepancy between the subjective level of disease-related information and objective knowledge of LDL-C treatment goals in a general CAD population. The majority of patients felt at least *well informed* about the topics of CAD, while less than 8% could name the correct LDL-C treatment goal. Additionally, we observed that knowledge of LDL-C goals was associated with goal attainment (Table 3). This finding represents a promising starting point for further investigations of disease-related patient knowledge and its interactions with the implementation of optimal secondary preventive pharmacotherapy. Prior positive evidence on associations between knowledge of HbA1c and glycemic control in DM patients underlines the potential of an informed patient for success in drug-modifiable risk factor control [19, 31]. Subsequently, further characterization of goal knowledge and associated factors is necessary. It should be clarified whether treatment goal knowledge results from effective patient‒physician risk factor communication (with good health information potentially leading to better adherence to lipid-lowering therapy) or whether it rather relates to the internal health locus of control in patients actively approaching their physician to improve risk factor control, among other factors [32]. 

The reporting of good adherence in 98.5% of patients assessed by the RAI requires cautious interpretation [16]. Data from other studies in CAD patients revealed high rates of nonadherence to cardiovascular pharmacotherapy [33]. Although the RAI is considered an established tool for assessing general adherence to pharmacotherapy, it relies on patient self-reports that might be inadequate [16, 34]. To overcome this limitation, novel methods, such as direct measurement of drug metabolites in urine, could contribute to objective measurements of drug adherence [35]. 

On the physician side, we observed a need for improvement in the prescription of effective lipid-lowering drugs in the management of dyslipidemia control. Less than one quarter of patients had been prescribed a combination therapy of statins and ezetimibe at the time of observation, despite low rates of goal attainment. These findings correspond to large real-world datasets from the United States and Europe [24, 32, 36]. Factors preventing physicians from prescribing guideline-recommended therapy to CAD patients remain to be further investigated. In the present study, risk factor management of LDL-C and HbA1c was performed predominantly by general practitioners (77%; Table 3). Two decades ago, in Germany, a voluntary, structured disease management program (DMP) for CAD patients was introduced by federal health institutions and bodies of the statutory health insurance. The DMP CAD aiming to improve secondary prevention and reduce health expenditures is predominantly coordinated by general practitioners with an estimated participation of 53–73% of all CAD patients [37]. Quality objectives of the DMP for CAD were last updated in 2019 [38]. Two parallel LDL-C-lowering strategies are promoted: Prescription of high-intensity statins for all patients or a goal directed-strategy with a goal of < 70 mg/dL for LDL-C [38]. Inconsistent LDL-C treatment goals set up among medical specialties reflect existing controversy about the quality of evidence supporting the lower LDL-C goals of the ESC [6, 39]. Possible uncertainty among physicians about the optimal LDL-C treatment goal for their patients might have influenced attainment and knowledge of treatment goals in this study.

Registry data from the United States identified cardiologist visits as a predictor of receiving intensified lipid-lowering therapy [40]. However, LDL-C measurement is infrequently performed by cardiologists (11%; Table 3), which may limit the impact of cardiologists. Interestingly, patients with DM were more likely to attain LDL-C treatment goals, which was possibly related to differences in management: Cardiovascular risk perceived by treating physicians may be greater in patients with DM and lead to focused attention to risk factor management. In Germany, diabetologists manage the treatment of DM in many patients and thus may also be a contributing factor to improved LDL-C risk factor control in patients with DM: LDL-C is a target of risk factor control in both CAD and DM care, which may increase the likelihood of receiving guideline-recommended therapy by any physician involved. The communication and interplay between cardiologists and general practitioners, including diabetologists in DM patients, must be the subject of further health services research. Limited evidence exists on differences in the adoption of guideline-recommended LDL-C treatment goals in CAD among different medical specialties in Germany [41]. Reasons previously identified for provider underuse of high-intensity statins, among others, are gaps in knowledge about statin benefits among physicians [42], discrepancies between LDL-C goals and generalist and specialist guidelines [43], beliefs about statin side effects [44], and clinical inertia [45]. Furthermore, a health care system-specific understanding of these reasons may help in the development of successful interventions aimed at the physician side.

### Limitations

This was a single-center cross-sectional study conducted in a tertiary care heart center. Its results are thus likely not to be extrapolated to other settings, e.g., ambulatory CAD patients in primary care. When compared to regional patients enrolled in primary care led DMP, the present population was younger (mean age 68 vs. 73 years) and had a higher proportion of male patients (75 vs. 65%) [37]. Prescription of ACE-inhibitors or angiotensin receptor-blockers (82 vs. 71%), betablockers (88 vs. 75%), and statins (91 vs. 85%) was recorded more frequently while DM as comorbidity (41 vs. 49%) was less frequently recorded [37]. However, with regards to mean age, proportion of male participants and use of preventive pharmacotherapy the present sample is comparable to CAD patients in the EU-wide DA VINCI registry on lipid-lowering therapy (mean age 67 years, 76% male, statins 94%) as well as to German CAD patients of the EUROASPIRE IV and V registries on cardiovascular risk factor control (mean age 69 years; 81–82% male, statins 87–97%, ACE-inhibitors or angiotensin receptor-blockers 86–93%, betablockers 79–87%) [24, 46]. The proportions of participants with DM (41%) were similar to DA VINCI (38%) and higher compared to EUROASPIRE IV and V registries (28–31%) [24, 46]. 

Thus, in this study we identified starting points for future research rather than explore the full range of complex interactions in the field of optimal secondary prevention of CAD (on both the patient and physician sides).

We did not further evaluate nonadherence to pharmacotherapy and were thus not able to determine how this could negatively influence treatment goal attainment; however, our results showed that adherence, as measured by the RAI, was higher than that reported in the current literature.

## Conclusion

In German CAD patients with and without DM presenting at a tertiary care center, a remarkable deficit in knowledge and attainment of ESC treatment goals of the drug-modifiable risk factor LDL-C exists compared with BP and HbA1c. DM comorbidity and patient knowledge were significantly associated with treatment goal attainment for LDL-C, showing potential for improvement through patient-centered as well as structural interventions.

## Electronic supplementary material

Below is the link to the electronic supplementary material.

Supplementary Material 1

Supplementary Material 2

Supplementary Material 3

## Data Availability

No datasets were generated or analysed during the current study.

## Declarations

### Patient and public involvement

The study was discussed with a patient who is a member of our cardiometabolic health services research group at HeinrichHeine University Düsseldorf.

## Abbreviations

ACE: Angiotensin converting enzyme
ASCVD: Atherosclerotic cardiovascular disease x
BP: Blood pressure
CAD: Coronary artery disease
DDP-4: Dipeptidyl peptidase-4
DM: Diabetes mellitus
DMP: Disease management program
HbA1c: Glycated hemoglobin
OR: Odds ratio
PCSK9: Proprotein convertase subtilisin/kexin type 9
RAI: Rief Adherence Index
SGLT2: Sodium‒glucose cotransporter 2
