# Supplementary material for: Associations of patient knowledge with drug-modifiable cardiovascular risk factor control in coronary artery disease patients with and without diabetes mellitus: results from the cross-sectional KNOW-ABC study
Source: BMC Cardiovasc Disord. 2025 Mar 5;25:148. doi: 10.1186/s12872-025-04599-7 (PMC11881313; doi:10.1186/s12872-025-04599-7)
Supplement: Supplementary file 1 — Supplementary Material 1 [file 12872_2025_4599_MOESM1_ESM.pdf]

Supplementary Table 1 – Treatment goal attainment, knowledge and subjective levels of information

Goal attainment	Total (n=204)	DM (n=84)	Without DM (n=120)	p-value
LDL-C	53 (26.0%)	33 (39.3%)	20 (16.7%)	**<0.01**
BP overall	147 (72.1%)	60 (71.4%)	87 (72.5%)	0.87
BP systolic	156 (76.5%)	5 (6.0%)	92 (76.7%)	0.94
BP diastolic	181 (88.7%)	75 (89.3%)	106 (88.3%)	0.83
HbA1c (<7.0%)	158 (77.5%)	41 (48.8%)	117 (97.5%)	-
HbA1c (<8.0 when ≥65 years)	177 (86.6%)	58 (69.0%)	119 (99.2%)	-
**Goal Knowledge**				
LDL-C	16 (7.8%)	5 (2.5%)	11 (9.2%)	0.83
BP overall	67 (32.8%)	31 (36.9%)	36 (30.0%)	0.30
BP systolic	113 (55.4%)	49 (58.3%)	64 (53.3%)	0.48
BP diastolic	79 (38.7%)	36 (42.9%)	43 (35.8%)	0.31
HbA1c	47 (23.0%)	45 (53.6%)	2 (1.7%)	-
**Summed subjective level of information**				
Topics of CAD	17.5 ±3	17.3 ±3	17.6 ±3	0.44

Supplementary Table 1: Attainment and patient knowledge of low-density lipoprotein cholesterol (LDL-C), blood pressure (BP), and glycated hemoglobin (HbA1c) treatment goals; summed subjective level of information on topics of CAD. Results of all included patients (n=204). Additionally, results of patients with (n=84) and without diabetes mellitus (DM, n=120) are reported separately. Data presented as n (%).

Supplementary Table 2 – Medical specialties: Risk factor management

Risk factor management	Total (n=204)	DM (n=84)	Without DM (n=120)	p-value
Measurement of LDL-C	195 (95.6%)	81 (96.4%)	114 (95.0%)	0.62
General physician	166 (85.1%)	72 (88.9%)	94 (82.5%)	0.21
Cardiologist	22 (11.2%)	5 (6.2%)	17 (14.9%)	0.06
Other specialty	7 (3.6%)	4 (4.9%)	3 (2.6%)	0.45
Measurement of HbA1c	117 (57.3%)	80 (95.2%)	37 (30.8%)	**<0.01**
General physician	90 (76.9%)	60 (75.0%)	30 (81.1%)	-
Cardiologist	10 (8.6%)	3 (3.8%)	7 (18.9%)	-
Other specialty	17 (14.5%)	17 (21.3%)	-	-

Supplementary Table 2: Risk factor management of low-density lipoprotein cholesterol (LDL-C) and glycated hemoglobin (HbA1c). Patients were asked to state which specialty of physician predominantly performed measurements of LDL-C and HbA1c. Results of all included patients (n=204). Additionally, results of patients with (n=84) and without diabetes mellitus (DM, n=120) are reported separately. Data presented as n (%).

Supplementary Table 3 – Medical Specialty: Patient attributed responsibility for risk factor control

Patient attributed responsibility	Total<br><br>(n=204)	DM<br><br>(n=84)	Without DM<br><br>(n=120)	p-value
LDL-C				
General physician	153 (75.0%)	64 (76.2%)	89 (74.2%)	0.74
Cardiologist	34 (16.7%)	11 (13.1%)	23 (19.2%)	0.25
Other specialty	7 (3.4%)	5 (6.0%)	2 (1.7%)	0.13
Patient	6 (2.9%)	3 (3.6%)	3 (2.5%)	0.69
Unknown	4 (2.0%)	1 (1.2%)	3 (2.5%)	0.64
HbA1c				
General physician	150 (73.5%)	57 (67.8%)	93 (77.5%)	0.12
Cardiologist	12 (5.9%)	4 (4.8%)	8 (6.7%)	0.57
Other specialty	20 (9.8%)	19 (22.6%)	1 (0.8%)	**<0.01**
Patient	3 (1.5%)	1 (1.2%)	2 (1.7%)	1.0
Unknown	19 (9.3%)	3 (3.6%)	16 (13.3%)	**0.03**

Supplementary Table 3: Patient attributed responsibility for risk factor management of low-density lipoprotein cholesterol (LDL-C) and glycated hemoglobin (HbA1c), with regard to medical specialty: Patients were asked to state who is responsible for management of LDL-C and HbA1c. Results of all included patients (n=204). Additionally, results of patients with (n=84) and without diabetes mellitus (DM, n=120) are reported separately. Data presented as n (%).

Supplementary Figure 1 - Subjective levels of disease-related information and information needs

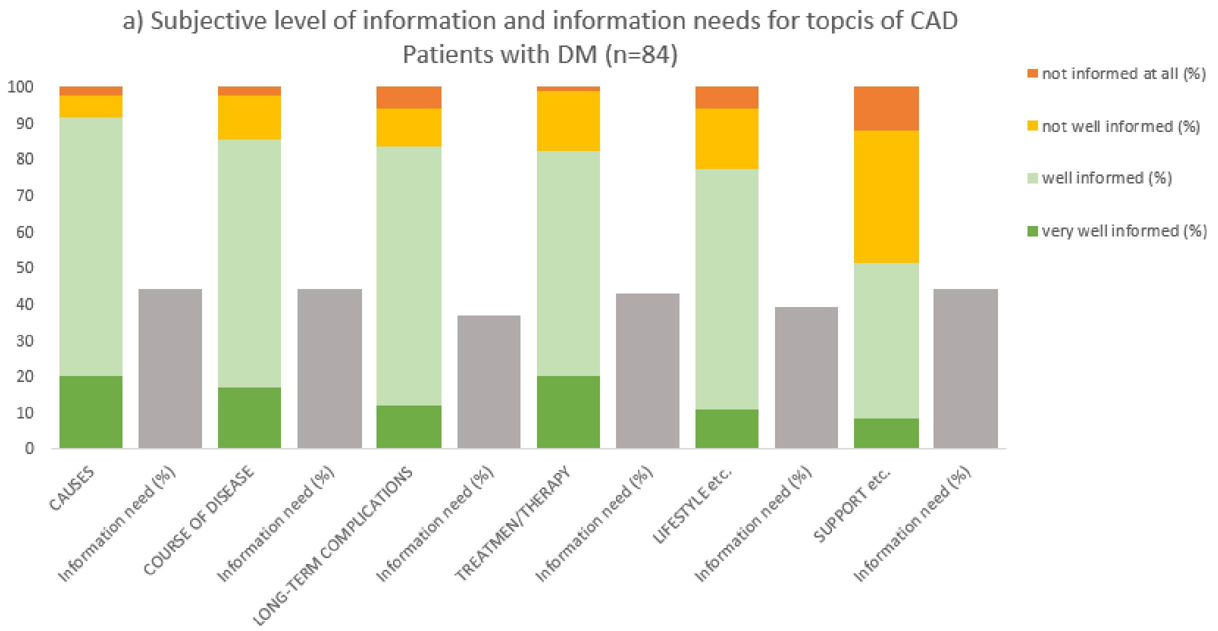

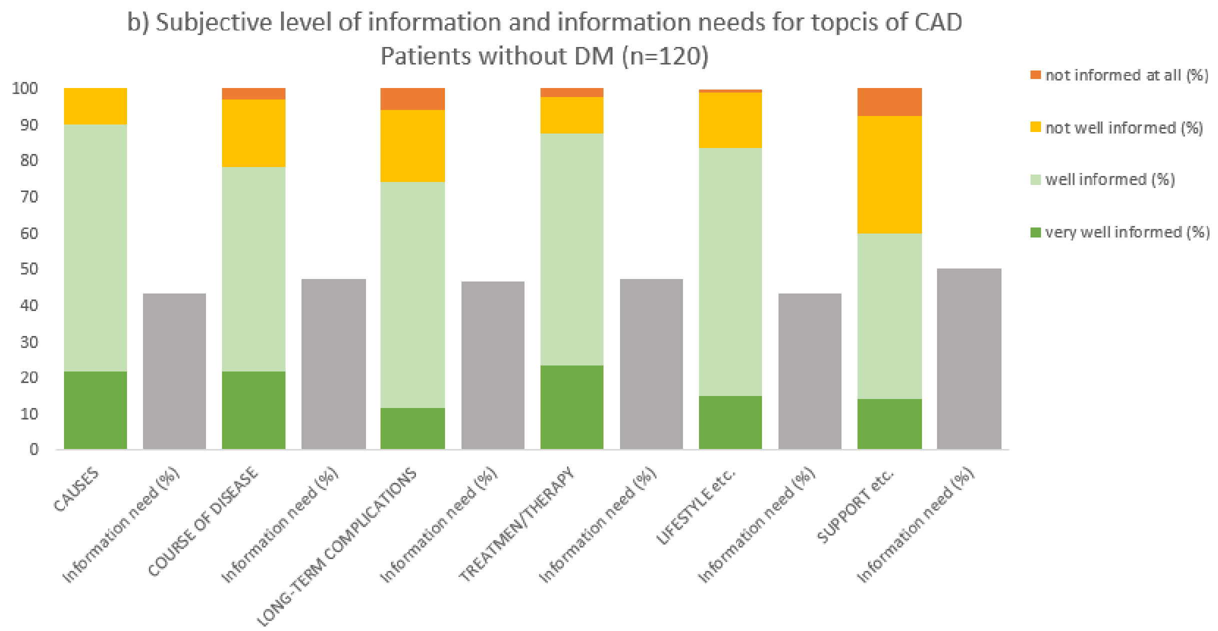

Supplementary Figure 1: Graphical display of subjective levels of disease-related information and subjective needs for more disease-related information on topics of coronary artery disease (CAD) in patients **a)** with (n=84) and **b)** without diabetes mellitus (DM; n=120). Subjective level of information was measured on a 4-point Likert-scale (*very well, well, not well, and not informed at all*). Additionally, patients were asked to state a need for additional information on every topic (*yes* or *no*). No significant differences were detected between patients with or without DM for all topics and respective information needs.
