# Supplementary material for: Associations of patient knowledge with drug-modifiable cardiovascular risk factor control in coronary artery disease patients with and without diabetes mellitus: results from the cross-sectional KNOW-ABC study
Source: BMC Cardiovasc Disord. 2025 Mar 5;25:148. doi: 10.1186/s12872-025-04599-7 (PMC11881313; doi:10.1186/s12872-025-04599-7)
Supplement: Supplementary file 2 — Supplementary Material 2 [file 12872_2025_4599_MOESM2_ESM.pdf]

QUESTIONNAIRE

1. Which **role** would you like to obtain **in treatment** of your disease. Please select one of the following.

- □ I prefer to make the final decision about which treatment I will receive.
- □ I prefer to make the final selection of my treatment after seriously considering my doctor's opinion.
- □ I prefer that my doctor and I share responsibility for deciding which treatment is best for me.
- □ I prefer that my doctor makes the final decision about which treatment will be used, but seriously considers my opinion.
- □ I prefer to leave all decisions regarding my treatment to my doctor.

2. When was your **coronary heart disease** diagnosed?

Please fill in here**:** ______________ years ago

OR **I don't know**: □

3. How well **informed** are you on the following **topics** of your **coronary heart disease**?

	**Would you currently like more information on the topic?**
**Causes of disease**	
□ very well □ well □ not well □ not informed at all	□ yes □ no
**Course of disease**	
□ very well □ well □ not well □ not informed at all	□ yes □ no
**Long-term complications**	
□ very well □ well □ not well □ not informed at all	□ yes □ no
**Treatment and therapy**	
□ very well □ well □ not well □ not informed at all	□ yes □ no
**Lifestyle adjustment, health promotion, and prevention**	
□ very well □ well □ not well □ not informed at all	□ yes □ no
**Support, helplines, and information sources**	
□ very well □ well □ not well □ not informed at all	□ yes □ no

4. Which value should your **LDL cholesterol** (also known as **‚bad cholesterol‘** or **LDL**) ideally **not exceed**?

Please state a **value:**______________ OR **I don’t know**: □

5. Have you ever been diagnosed with **high blood pressure**?

Yes □ **No** □

If yes, when was the diagnosis made?

______________ years ago OR **I don’t know**: □

6. How well **informed** are you on the following **topics** of your **high blood pressure**?

	**Would you currently like more information on the topic?**
**Causes of disease**	
□ very well □ well □ not well □ not informed at all	□ yes □ no
**Course of disease**	
□ very well □ well □ not well □ not informed at all	□ yes □ no
**Long-term complications**	
□ very well □ well □ not well □ not informed at all	□ yes □ no
**Treatment and therapy**	
□ very well □ well □ not well □ not informed at all	□ yes □ no
**Lifestyle adjustment, health promotion, and prevention**	
□ very well □ well □ not well □ not informed at all	□ yes □ no
**Support, helplines, and information sources**	
□ very well □ well □ not well □ not informed at all	□ yes □ no

7. Which value should your **blood pressure** ideally **not exceed**?

Please fill in here**:** Upper value_______/lower value_______ OR **I don’t know**: □

8. Have you ever been diagnosed with **diabetes mellitus**?

Yes □ **No** □

If yes, when was the diagnosis made?

______________ years ago OR **I don't know**: □

9. How well **informed** are you on the following **topics** of your **diabetes mellitus**?

	**Would you currently like more information on the topic?**
**Causes of disease**	
□ very well □ well □ not well □ not informed at all	□ yes □ no
**Course of disease**	
□ very well □ well □ not well □ not informed at all	□ yes □ no
**Long-term complications**	
□ very well □ well □ not well □ not informed at all	□ yes □ no
**Treatment and therapy**	
□ very well □ well □ not well □ not informed at all	□ yes □ no
**Lifestyle adjustment, health promotion, and prevention**	
□ very well □ well □ not well □ not informed at all	□ yes □ no
**Support, helplines, and information sources**	
□ very well □ well □ not well □ not informed at all	□ yes □ no

10. Which value should your **HbA1c** in % (also known as **long-term blood glucose value**) ideally **not exceed**?

Please state a **value:**______________ OR **I don't know**: □

11. If you consider all past behaviors related to **prescribed medications**, how often have you done the following?

	**almost never happened (in 0-20 % of cases)**	**rarely happened (in 20-40 % of cases)**	**often happened (in 40-60 % of cases)**	**happened most of the time (in 60-80 %)**	**almost always happened (in 80-100 % of cases)**
I stored or threw away prescribes medication without unwrapping it	□ 1	□ 2	□ 3	□ 4	□ 5
I changed the doses of my medication without doctor's authorisation depending on my well-being	□ 1	□ 2	□ 3	□ 4	□ 5
I discontinued my medication earlier then the doctor recommended	□ 1	□ 2	□ 3	□ 4	□ 5
I discontinued my medication because of mild side-effects	□ 1	□ 2	□ 3	□ 4	□ 5

12. Who carried out the majority of the **LDL cholesterol measurements**? Please select **one** of the following options.

no measurements have been performed □

general physician □ **cardiologist** □ **other specialty** □

OR **I don't know**: □

13. Who do you think is **responsible** for **adjusting medication** and **controlling LDL cholesterol**? Please select **one** of the following options.

patient □ **general physician** □ **cardiologist** □

other specialty □ OR **I don’t know**: □

14. Who carried out the majority of the **HbA1c measurements**? Please select **one** of the following options.

no measurements have been performed □

general physician □ **cardiologist** □ **other specialty** □

OR **I don’t know**: □

15. Who do you think is mainly **responsible** for **adjusting medication** and **controlling HbA1c**? Please select **one** of the following options.

patient □ **general physician** □ **cardiologist** □

other specialty □ OR **I don’t know**: □

16. What is your **highest level of education**?

□ no degree
□ main/public school
□ middle school
□ high school diploma
□ university degree
